# Retraction for Atractylenolide I enhances responsiveness to immune checkpoint blockade therapy by activating tumor antigen presentation

**DOI:** 10.1172/JCI202473

**Published:** 2026-01-16

**Authors:** Hanchen Xu, Kevin Van der Jeught, Zhuolong Zhou, Lu Zhang, Tao Yu, Yifan Sun, Yujing Li, Changlin Wan, Ka Man So, Degang Liu, Michael Frieden, Yuanzhang Fang, Amber L. Mosley, Xiaoming He, Xinna Zhang, George E. Sandusky, Yunlong Liu, Samy O. Meroueh, Chi Zhang, Aruna B. Wijeratne, Cheng Huang, Guang Ji, Xiongbin Lu

Original citation: *J Clin Invest*. 2021;131(10):e146832. https://doi.org/10.1172/JCI146832

Citation for this retraction: *J Clin Invest*. 2026;136(1):e202473. https://doi.org/10.1172/JCI202473

Indiana University, The Ohio State University, and the University of Maryland, College Park jointly notified the *JCI* of data falsification in Figures 2B and 6D and misconduct findings against Xiongbin Lu and Hanchen Xu. In accordance with the investigation committee’s recommendation, the *JCI* is retracting this article.

An independent investigation by Longhua Hospital, Shanghai University of Traditional Chinese Medicine is ongoing.

Hanchen Xu, Zhuolong Zhou, Xiaoming He, Xinna Zhang, and Xiongbin Lu dissent from the Journal’s decision to retract. The remaining authors abstained from commenting or could not be reached.

## Version 1 01/16/2026

Electronic publication

